# Supplementary material for: Common and distinct contributions of the dorsomedial prefrontal and posterior parietal cortices to social episodic memory
Source: Soc Cogn Affect Neurosci. 2026 Aug 25;21(1):nsag070. doi: 10.1093/scan/nsag070 (PMC13592675; doi:10.1093/scan/nsag070)
Supplement: Supplementary file 1 [file nsag070_supplementary_data.docx]

**Supplementary Section**

**Supplementary 1: Encoding and Retrieval RTs**

A mixed repeated-measures ANOVA was conducted to examine the effects of Agent (Self, Friend, Celebrity; within-subjects), Stimulation (two levels; between-subjects), and Region (two levels; between-subjects) on encoding time. Mauchly's test indicated that the assumption of sphericity was violated; therefore, Greenhouse–Geisser corrections were applied to the within-subjects effects.

There was a significant main effect of Agent, *F*(1.76, 168.61) = 21.23, *p* < .001, ηp² = .181, indicating that encoding time differed across the three agent conditions. Holm-adjusted pairwise comparisons showed that encoding time did not differ significantly between the Self and Friend conditions (*p* = .849). However, participants took significantly longer to encode Celebrity stimuli than both Self stimuli (mean difference = 0.56, *p* < .001) and Friend stimuli (mean difference = 0.54, *p* < .001).

There was also a significant Agent × Stimulation interaction, *F*(1.76, 168.61) = 3.44, *p* = .040, ηp² = .035, indicating that the effect of stimulus condition on encoding time differed across agent conditions. However, the Agent × Region interaction was not significant, *F*(1.76, 168.61) = 0.41, *p* = .637, ηp² = .004, nor was the three-way Agent × Stimulation × Region interaction, *F*(1.76, 168.61) = 1.91, *p* = .156, ηp² = .020. No significant between-subjects effects were observed for Stimulation, Region, or the Stimulation × Region interaction (all *ps* > .29).

To investigate the significant Agent × Stimulation interaction, simple effects analyses were conducted separately for each agent condition. For self-related encoding time, an independent-samples Student's *t*-test showed no significant difference between the two stimulus groups, *t*(98) = −0.17, *p* = .867. Similarly, no significant difference was observed for friend-related encoding time, *t*(98) = 1.14, *p* = .255. Because the assumptions of parametric testing were violated for celeb-related encoding time, a Mann–Whitney *U* test was conducted, which also indicated no significant difference between stimulus groups, *U* = 1453.00, *p* = .163.

Overall, participants required significantly longer to encode celebrity-related information than self- or friend-related information, whereas encoding times for self- and friend-related stimuli did not differ. Although the Agent × Stimulation interaction reached statistical significance, follow-up simple effects analyses did not reveal significant differences between stimulation groups within any individual agent condition.

Encoding response times had no association with subsequent memory sensitivity or metacognitive efficiency for Self, r=0.09, p=.54 & 0.06, p=.66 or Friend, r=.16, p=.26 & r=0.15, p=.28. However, encoding response times did predict memory sensitivity for Celebrity encoded words, r=0.36, p=0.01, but not memory efficiency, r= -0.04, p=.79.

Crucially, encoding response times were consistent across stimulation conditions for Self, t(98)= -0.17, p= .87, d= -0.03, Friend, t(98)= 1.14, p= .26, d= 0.23, and Celebrity (Mann-Whitney due to a violation of the assumption of equal variances), t(98)= 1453, p= .16, r_rb_= -0.16.

**Supplementary Table 1.** *Descriptive statistics for encoding and retrieval response times.*

| Agent | Stimulation | Region | *Encoding* | | | | *Retrieval* | | |
| --- | --- | --- | --- | --- | --- | --- | --- | --- | --- |
|  |  |  | *n* | *M* | *SD* | *Range* | *M* | *SD* | *Range* |
| Self | Sham | DmPFC | 25 | 3.96 | 0.99 | 2.14-6.33 | 2.97 | 0.99 | 0.31-5.08 |
| Self | Sham | PPC | 25 | 4.26 | 1.59 | 2.24-9.41 | 2.78 | 1.09 | 1.71-6.51 |
| Self | Active | DmPFC | 25 | 4.10 | 1.32 | 2.31-8.23 | 3.02 | 0.93 | 1.73-5.08 |
| Self | Active | PPC | 25 | 4.21 | 0.94 | 2.72-6.35 | 2.55 | 0.86 | 1.20-5.11 |
| Friend | Sham | DmPFC | 25 | 4.11 | 1.22 | 2.05-6.65 | 2.83 | 0.71 | 1.11-4.16 |
| Friend | Sham | PPC | 25 | 4.48 | 1.85 | 2.39-11.23 | 2.70 | 0.84 | 1.70-4.60 |
| Friend | Active | DmPFC | 25 | 3.88 | 1.05 | 2.21-6.78 | 3.03 | 0.79 | 1.94-5.12 |
| Friend | Active | PPC | 25 | 4.11 | 0.88 | 2.71-5.76 | 2.65 | 0.71 | 1.26-4.32 |
| Celebrity | Sham | DmPFC | 25 | 4.65 | 1.09 | 2.17-6.29 | 2.79 | 0.92 | 0.53-5.06 |
| Celebrity | Sham | PPC | 25 | 5.19 | 1.74 | 2.73-8.62 | 2.69 | 0.78 | 1.61-4.53 |
| Celebrity | Active | DmPFC | 25 | 4.61 | 1.29 | 2.22-8.15 | 3.07 | 0.83 | 1.82-4.91 |
| Celebrity | Active | PPC | 25 | 4.31 | 0.83 | 2.90-6.85 | 2.54 | 0.80 | 1.34-4.87 |

A 3 (Agent: Self, Friend, Celebrity) × 2 (Stimulation: Active, Sham) × 2 (Region) mixed repeated-measures ANOVA was conducted to examine the effects of Agent, Stimulation, and Region on retrieval response time. There was no significant main effect of Agent, *F*(2, 192) = 0.42, *p* = .655, indicating that retrieval response times did not differ significantly across the self-, friend-, and celebrity-related conditions.

The Agent × Region interaction was not significant, *F*(2, 192) = 0.19, *p* = .828, indicating that the effect of stimulation region on retrieval response time did not differ across agent conditions. Similarly, the Agent × Stimulation interaction was not significant, *F*(2, 192) = 1.14, *p* = .321, suggesting that stimulation did not differentially influence retrieval response time for self-, friend-, or celebrity-related stimuli. The three-way Agent × Region × Stimulation interaction was also not significant, *F*(2, 192) = 0.28, *p* = .758.

There were no significant between-subjects effects. Neither the main effect of Region, *F*(1, 96) = 3.64, *p* = .059, nor the main effect of Stimulation, *F*(1, 96) = 0.01, *p* = .922, reached statistical significance. Likewise, the Region × Stimulation interaction was not significant, *F*(1, 96) = 1.05, *p* = .309.

Overall, these findings indicate that retrieval response time was not significantly influenced by agent, stimulation, stimulation region, or any interactions between these factors.

Correlations between retrieval response times and memory sensitivity and metacognitive efficiency were all non-significant (p’s between 0.29-0.96).

Total encoding times were between 127.26 and 585.12 seconds, with all participants completing during active stimulation (1200 seconds in duration).

A two-way between-subjects ANOVA was conducted to examine the effects of Stimulation (Active vs. Sham) and Region on overall encoding time. There was no significant main effect of Stimulation, *F*(1, 96) = 1.09, *p* = .298, ηp² = .011, indicating that overall encoding time did not differ between the active and sham stimulation conditions. Likewise, there was no significant main effect of Region, *F*(1, 96) = 0.84, *p* = .362, ηp² = .009, suggesting that overall encoding time did not differ between stimulation regions. Furthermore, the Stimulation × Region interaction was not significant, *F*(1, 96) = 0.71, *p* = .400, ηp² = .007, indicating that the effect of stimulation on overall encoding time did not vary as a function of stimulation region.

**Supplementary 2: Full analyses for item memory**

*Sensitivity (d’)*

Repeated-Measures ANOVA Results

| Effect | df | F | p | η²G |
| --- | --- | --- | --- | --- |
| Agent | 2, 192 | 167.61 | < .001 | .213 |
| Agent × Region | 2, 192 | 2.27 | .107 | .004 |
| Agent × Stim | 2, 192 | 6.82 | .001 | .011 |
| Agent × Region × Stim | 2, 192 | 0.62 | .540 | .001 |
| Valence | 1, 96 | 2.71 | .103 | .004 |
| Valence × Region | 1, 96 | 3.15 | .079 | .005 |
| Valence × Stim | 1, 96 | 0.01 | .936 | < .001 |
| Valence × Region × Stim | 1, 96 | 0.01 | .926 | < .001 |
| Agent × Valence | 2, 192 | 0.39 | .680 | .001 |
| Agent × Valence × Region | 2, 192 | 0.99 | .373 | .002 |
| Agent × Valence × Stim | 2, 192 | 0.10 | .905 | < .001 |
| Agent × Valence × Region × Stim | 2, 192 | 0.27 | .767 | < .001 |
| Region | 1, 96 | 0.10 | .755 | < .001 |
| Stim | 1, 96 | 0.37 | .545 | .002 |
| Region × Stim | 1, 96 | 0.06 | .808 | < .001 |

Note. Type III sums of squares were used. η²G = generalized eta squared.

*Metacognitive Sensitivity (meta d’/d’)*

Repeated-Measures ANOVA Results

| Effect | df | F | p | η²G |
| --- | --- | --- | --- | --- |
| Agent | 1.84, 173.02 | 84.27 | < .001 | .128 |
| Agent × Stim | 1.84, 173.02 | 0.56 | .558 | .001 |
| Agent × Region | 1.84, 173.02 | 4.73 | .012 | .008 |
| Agent × Stim × Region | 1.84, 173.02 | 0.33 | .699 | .001 |
| Valence | 1, 94 | 0.53 | .469 | .001 |
| Valence × Stim | 1, 94 | 0.01 | .913 | < .001 |
| Valence × Region | 1, 94 | 21.45 | < .001 | .026 |
| Valence × Stim × Region | 1, 94 | 0.04 | .836 | < .001 |
| Agent × Valence | 1.89, 177.39 | 24.57 | < .001 | .032 |
| Agent × Valence × Stim | 1.89, 177.39 | 0.12 | .880 | < .001 |
| Agent × Valence × Region | 1.89, 177.39 | 2.23 | .113 | .003 |
| Agent × Valence × Stim × Region | 1.89, 177.39 | 0.82 | .438 | .001 |
| Stim | 1, 94 | 2.13 | .148 | .013 |
| Region | 1, 94 | 0.54 | .464 | .003 |
| Stim × Region | 1, 94 | 2.88 | .093 | .018 |

Note. Type III sums of squares were used. Greenhouse–Geisser–corrected degrees of freedom are reported where Mauchly’s test indicated violation of sphericity. η²G = generalized eta squared.

***Supplementary 3: Full analyses for source memory***

*Source Memory (d’)*

Repeated-Measures ANOVA Results

| Effect | df | F | p | η²G |
| --- | --- | --- | --- | --- |
| Agent | 2, 192 | 20.23 | < .001 | .044 |
| Agent × Stim | 2, 192 | 1.67 | .191 | .004 |
| Agent × Region | 2, 192 | 0.48 | .619 | .001 |
| Agent × Stim × Region | 2, 192 | 0.14 | .872 | < .001 |
| Valence | 1, 96 | 0.77 | .382 | .002 |
| Valence × Stim | 1, 96 | 1.42 | .237 | .003 |
| Valence × Region | 1, 96 | 2.26 | .136 | .005 |
| Valence × Stim × Region | 1, 96 | 4.78 | .031 | .010 |
| Agent × Valence | 2, 192 | 20.67 | < .001 | .038 |
| Agent × Valence × Stim | 2, 192 | 0.11 | .897 | < .001 |
| Agent × Valence × Region | 2, 192 | 1.11 | .333 | .002 |
| Agent × Valence × Stim × Region | 2, 192 | 1.06 | .347 | .002 |
| Stim | 1, 96 | 0.49 | .488 | .002 |
| Region | 1, 96 | 0.00 | .970 | < .001 |
| Stim × Region | 1, 96 | 1.18 | .279 | .005 |

Note. Type III sums of squares were used. η²G = generalized eta squared.

*Metacognitive Efficiency (Meta d’/d’)*

Repeated-Measures ANOVA Results

| Effect | df | F | p | η²G |
| --- | --- | --- | --- | --- |
| Agent | 2, 176 | 47.42 | < .001 | .074 |
| Agent × Stim | 2, 176 | 0.70 | .496 | .001 |
| Agent × Region | 2, 176 | 3.32 | .038 | .006 |
| Agent × Stim × Region | 2, 176 | 1.44 | .240 | .002 |
| Valence | 1, 88 | 277.97 | < .001 | .475 |
| Valence × Stim | 1, 88 | 0.11 | .740 | < .001 |
| Valence × Region | 1, 88 | 5.59 | .020 | .018 |
| Valence × Stim × Region | 1, 88 | 0.00 | .987 | < .001 |
| Agent × Valence | 2, 176 | 1.76 | .176 | .003 |
| Agent × Valence × Stim | 2, 176 | 0.76 | .467 | .001 |
| Agent × Valence × Region | 2, 176 | 6.53 | .002 | .012 |
| Agent × Valence × Stim × Region | 2, 176 | 1.04 | .357 | .002 |
| Stim | 1, 88 | 0.18 | .674 | .001 |
| Region | 1, 88 | 0.04 | .841 | < .001 |
| Stim × Region | 1, 88 | 0.01 | .925 | < .001 |

Note. Type III sums of squares were used. η²G = generalized eta squared. Where Mauchly’s test indicated violation of sphericity, corrected statistics were used.

**Supplementary 3: Mood and adverse effects**

To assess whether stimulation influenced mood or tolerability, separate two-way ANOVAs were conducted on change scores for negative mood, positive mood, and total adverse effects. Each analysis included Region (PPC vs. dmPFC) and Stimulation (active vs. sham) as between-subjects factors. Means and standard deviations are presented in Table 3.

For negative mood change, there was no significant main effect of Region, *F*(1, 96) = 1.33, *p* = .25, η²p = .014, no main effect of Stimulation, *F*(1, 96) = 0.00, *p* = .95, η²p < .001, and no Region × Stimulation interaction, *F*(1, 96) = 0.82, *p* = .37, η²p = .009.

Similarly, for positive mood change, there were no significant main effects of Region, *F*(1, 96) = 0.03, *p* = .86, η²p < .001, or Stimulation, *F*(1, 96) = 0.36, *p* = .55, η²p = .004, and no Region × Stimulation interaction, *F*(1, 96) = 0.65, *p* = .42, η²p = .007.

Analysis of total adverse effects scores revealed no significant main effect of Region, *F*(1, 96) = 0.00, *p* = 1.00, η²p < .001, no main effect of Stimulation, *F*(1, 96) = 0.46, *p* = .50, η²p = .005, and no Region × Stimulation interaction, *F*(1, 96) = 1.62, *p* = .21, η²p = .017.

Together, these analyses indicate that stimulation did not differentially affect mood or adverse effects across regions or stimulation conditions, suggesting that the observed memory effects are unlikely to be attributable to changes in affect or tolerability.

**Supplementary Table 2.** *Descriptive statistics (mean ± SD) for mood change scores and total adverse effects by stimulation site and condition.*

|  | PPC – Sham  (n = 25) | PPC – Active  (n = 25) | dmPFC – Sham (n = 25) | dmPFC – Active (n = 25) |
| --- | --- | --- | --- | --- |
| Negative mood | −2.64 ± 6.65 | −1.56 ± 5.84 | −0.35 ± 5.63 | −1.28 ± 3.71 |
| Positive mood | −0.30 ± 4.41 | −1.23 ± 2.37 | −0.72 ± 3.27 | −0.58 ± 2.94 |
| Adverse effects | 3.24 ± 3.18 | 2.20 ± 3.15 | 2.56 ± 1.85 | 2.88 ± 2.24 |

*Note.* Mood change scores were calculated as post- minus pre-stimulation composite scores. Negative mood reflects the summed ratings of afraid, confused, sad, angry, tired, and tense, and positive mood reflects the summed ratings of energetic and happy. Total adverse effects scores represent the sum across all adverse effects items (maximum score = 40).

**Supplementary 4: Blinding**

A chi-square test of independence was conducted to examine whether guessing accuracy differed across the two regions. Accuracy (correct vs. incorrect) was significantly associated with region, χ²(1, *N* = 100) = 7.89, *p* = .005. Participants produced a higher proportion of correct guesses at the dmPFC (34/50; 68%) compared with the PPC (20/50; 40%).

To assess performance relative to chance, binomial tests were conducted separately for each region (chance = 0.5). At the dmPFC, the proportion of correct guesses was significantly greater than chance (34/50; *p* = .02). In contrast, performance in the PPC group did not differ significantly from chance (20/50; *p* = .89).
